# Cognitive and Neuroplasticity Mechanisms by Which Congenital or Early Blindness May Confer a Protective Effect Against Schizophrenia

**DOI:** 10.3389/fpsyg.2012.00624

**Published:** 2013-01-21

**Authors:** Steven M. Silverstein, Yushi Wang, Brian P. Keane

**Affiliations:** ^1^University Behavioral HealthCare, University of Medicine and Dentistry of New JerseyPiscataway, NJ, USA; ^2^Department of Psychiatry, University of Medicine and Dentistry of New Jersey, Robert Wood Johnson Medical SchoolPiscataway, NJ, USA; ^3^Rutgers University Center for Cognitive SciencePiscataway, NJ, USA

**Keywords:** schizophrenia, blindness, perception, cognition, vision, vision disorders, plasticity

## Abstract

Several authors have noted that there are no reported cases of people with schizophrenia who were born blind or who developed blindness shortly after birth, suggesting that congenital or early (C/E) blindness may serve as a protective factor against schizophrenia. By what mechanisms might this effect operate? Here, we hypothesize that C/E blindness offers protection by strengthening cognitive functions whose impairment characterizes schizophrenia, and by constraining cognitive processes that exhibit excessive flexibility in schizophrenia. After briefly summarizing evidence that schizophrenia is fundamentally a cognitive disorder, we review areas of perceptual and cognitive function that are both impaired in the illness and augmented in C/E blindness, as compared to healthy sighted individuals. We next discuss: (1) the role of neuroplasticity in driving these cognitive changes in C/E blindness; (2) evidence that C/E blindness does not confer protective effects against other mental disorders; and (3) evidence that other forms of C/E sensory loss (e.g., deafness) do not reduce the risk of schizophrenia. We conclude by discussing implications of these data for designing cognitive training interventions to reduce schizophrenia-related cognitive impairment, and perhaps to reduce the likelihood of the development of the disorder itself.

## Introduction

Over the past 60 years, several authors (Chevigny and Braverman, 1950; Abely and Carton, 1967; Horrobin, 1979; Riscalla, 1980; Feierman, 1982; Sanders et al., 2003) have postulated that congenital or early (C/E) blindness may serve as a protective factor against the development of schizophrenia. Supporting data derive from several sources, including literature reviews, examination of cohorts of blind patients in psychiatric hospitals, and surveys of agencies that treat large numbers of blind people. The most recent of these Sanders et al. (2003) noted that across all past papers, there has not been even one reported case of a congenitally blind person who developed schizophrenia. While we identified one reported case of a blind “schizophrenic” child (Stewart and Sardo, 1965), the 6-year-old described in that paper had many symptoms of autism and no symptoms of psychosis, and would almost certainly be diagnosed with an autism-spectrum disorder using DSM-III (American Psychiatric Association, 1980) or later criteria. In contrast to the lack of cases of people with C/E blindness who meet modern criteria for schizophrenia, reports do exist of people who develop both blindness and schizophrenia later in life (Checkley and Slade, 1979). We therefore hypothesize that it is the brain changes that occur secondary to C/E blindness – rather than blindness *per se* – that protect against schizophrenia. However, the mechanisms that confer this apparent protection have not yet been identified. In this paper, we advance the hypothesis that protection occurs by (1) strengthening the perceptual and cognitive functions whose impairment forms the essential nature of schizophrenia; and (2) constraining cognitive processes that are excessively neuroplastic in the illness.

In the discussion below, after briefly summarizing the position that schizophrenia is fundamentally a cognitive (i.e., information-processing) disorder, we review evidence that a cluster of perceptual and cognitive functions is at once markedly impaired in schizophrenia and significantly augmented in C/E blindness, compared to healthy sighted individuals (see Table 1). In subsequent sections, we explicate the role of neuroplasticity in driving these perceptual and cognitive differences, we provide evidence that C/E blindness does not confer protection against other mental disorders (e.g., depression, anorexia nervosa) and finally we argue that other forms of C/E sensory loss (e.g., deafness) do not reduce the risk of schizophrenia. We conclude by noting the implications of these data for designing cognitive training interventions for patients and people at high risk for the disorder, and for developing interventions to prevent schizophrenia.

**Table 1:** **Summary of cognitive and brain function enhancements (+) and impairments (−), compared to healthy sighted individuals, in C/E blindness and schizophrenia**.

Function	Blindness	Schizophrenia
**AUDITORY PERCEPTION**		
Localization	+	−
Acuity	+	−
Discrimination	+	−
Comprehension	+	−
Categorization	+	−
Temporal resolution	+	−
Latency of auditory ERPs	+*	−**
**AUDITORY ATTENTION**		
Preattentive processing (e.g., MMN)	+	−
Selective attention	+	−
Divided attention	+	−
**MEMORY**		
Working memory	+	−
Short-term memory	+	−
Long-term memory	+	−
**LANGUAGE**		
Lexical decision making	+*	−**
Abstraction	−	+
Conceptual inclusiveness	−	+
Word inventions	−	+
**CONSTRUCTION OF SUBJECTIVE EXPERIENCE**		
Integration via serial processing	+	−
Holistic processing	+	−
Olfaction	+	−
Motor control	+	−
Body perception	+	−
Plasticity	+	−

*Faster than normal.

**Slower than normal.

## Schizophrenia as a Cognitive Disorder: Kraepelin and Dementia Praecox Revisited

Although the most obvious clinical features of schizophrenia include psychotic symptoms such as hallucinations, delusions, and bizarre behavior, it has long been thought that such symptoms are secondary, compensatory, features of the disorder. In contrast, much evidence suggests that the core features of schizophrenia are cognitive in nature, involving disturbances in perception, attention, memory, learning, language, and context-based modulation of these processes as a function of expectations and action plans (Kraepelin, 1903; Bleuler, 1950; Weiss, 1989; Phillips and Silverstein, 2003; Nuechterlein et al., 2012; Tamminga, 2013; Khan, in press). In this way, schizophrenia can be viewed as similar to brain disorders such as Parkinson’s disease dementia and dementia with Lewy bodies, in which psychotic symptoms can occur, but are often late-developing phenomena, and are not considered the defining features of the disorder (Ballard et al., 2001; Williams-Gray et al., 2006; Ffytche, 2007). A large body of research evidence now indicates that perceptual and cognitive impairments are found in nearly all people with schizophrenia, and occur with greater frequency than psychotic symptoms (Palmer et al., 2009). Significant cognitive impairment is also easily detectable in most schizophrenia patients even when psychotic symptoms are in remission (Altshuler et al., 2004). In addition: (1) low intelligence is a risk factor for schizophrenia; (2) cognitive decline and intellectual underperformance precede the onset of psychotic symptoms (and therefore the diagnosis) by many years; (3) decline in cognitive functioning continues after psychosis onset; and (4) this pattern of changes is not found in other major mental illnesses, even those that can include psychotic symptoms, such as bipolar disorder (Neumann et al., 1995; Schenkel and Silverstein, 2004; Khan, in press). Cognitive impairments in schizophrenia are also significantly correlated with level of functional disability (Green, 1996; Silverstein et al., 1998), and are therefore important treatment targets.

## Domains of Perceptual and Cognitive Functioning that are Strengthened by C/E Blindness and Impaired in Schizophrenia

### Auditory perception

Compared to sighted people (and in some cases to late-blind people), C/E blind individuals are characterized by enhanced auditory acuity for pitch, better pitch discrimination, better pitch-timbre categorization (Wan et al., 2010), better speech discrimination (Hertrich et al., 2009), better sound localization, better temporal auditory resolution, better noise-embedded speech discrimination (Niemeyer and Starlinger, 1981; Muchnik et al., 1991), lower sound threshold for identification of pseudo-words (Rokem and Ahissar, 2009), shorter latencies for auditory event-related potentials (ERPs; Roder et al., 1996, 1999), and greater tolerance for short stimulus-onset asynchronies in an auditory backward masking task (Stevens and Weaver, 2005). C/E blindness is also associated with comprehension of ultra-fast synthetic speech at a rate of ∼25 syllables per second, which is close to three times the rate at which non-blind individuals can comprehend such speech (Hertrich et al., 2009). In addition, C/E blind subjects have shown larger mismatch negativity (MMN) components of the ERP, suggesting a compensatory improvement in pre-attentional processes (Kujala et al., 1995). It has been demonstrated that these sensory and perceptual processing differences are due to enhanced basic perceptual skills, not to “higher order” functions such as attention, memory, language, or executive functions (reviewed in Cattaneo and Vecchi, 2011).

The above findings are in contrast to what has been observed in schizophrenia, in which, for example, patients are typically characterized by poor auditory acuity, precision, and discrimination (including in tone matching) (Javitt et al., 1997; Rabinowicz et al., 2000; Li et al., 2002; Rojas et al., 2007; Turetsky et al., 2009; Perrin et al., 2010; Kantrowitz et al., 2013), impairments in sound localization (Perrin et al., 2010), temporal resolution of sound (Foucher et al., 2007), auditory backward masking (Kallstrand et al., 2002), pre-attentional processing as indicated by the (smaller) MMN ERP component (Umbricht et al., 2003), and longer latency of post-attentional auditory ERPs (Ward et al., 1991; Sevik et al., 2011; Iyer et al., 2012). Individuals with schizophrenia also have abnormal processing of speech sounds (Ngan et al., 2003; Hirano et al., 2008), are worse than other groups at detecting speech embedded in noise, and are more likely than controls to identify non-speech sounds as speech (Vercammen et al., 2008). Importantly, it has been shown that these impairments in pitch processing in schizophrenia are not secondary to symptoms, medication, or poor attentional functioning (Rabinowicz et al., 2000).

The impaired auditory functions noted above are not mere laboratory curiosities; they have important clinical and functional implications. For example, poor sound localization is thought to be associated with an externalization bias in which there is a reduced ability to recognize that the sound of one’s own voice is coming from the self, as opposed to an external cause, which may contribute to delusional thinking (Johns et al., 2001; Ilankovic et al., 2011). At a more general level, perception has been conceptualized as an active, hypothesis-generating function that involves generating a model of the world, and then updating this model via coding of prediction error (i.e., the degree to which continued incoming sensory input does not conform to the current perceptual model of the world; Corlett et al., 2009; Clark, in press). It has been postulated that symptoms of schizophrenia, such as hallucinations and delusions may result from an impaired ability to detect inconsistencies between what would normally be expected (based on past experience) and incoming sensory information (Corlett et al., 2007, 2009; Clark, in press). Both faulty bottom-up processing and poor quality top-down feedback, as have been demonstrated in schizophrenia (Silverstein and Keane, 2009, 2011a,b), would degrade the quality of perceptual representations and the extent to which they are modified by context to most accurately reflect the nature of reality (Corlett et al., 2007, 2009; Clark, in press; Silverstein, in press). The result can be abnormal perceptions, hypersalience of normally ignored stimuli, and new and unusual beliefs to account for these changes in subjective experience – in short, an internal representation of the world that can deviate markedly from reality, and increasingly so over time (Clark, in press). In contrast, as C/E blindness is associated with enhanced sensory and perceptual processing, it can be seen how this would reduce the likelihood of the sort of prediction coding failures, and their sequelae, associated with schizophrenia. The auditory impairments in schizophrenia described above have also been shown to have other important consequences, which would not occur in people with C/E blindness, such as problems decoding cues to emotion in speech (Leitman et al., 2011; Gold et al., 2012) and impaired semantic analysis (Leitman et al., 2007).

### Auditory attention

Blindness has been associated with enhancements in several components of attention that are impaired in schizophrenia. For example, on reaction time tasks, blind subjects are superior to sighted individuals in responding to auditory and tactile cues (Collignon and De Volder, 2009). On tactile and auditory *selective* attention tasks involving spatial discrimination, blind people are more efficient than sighted subjects (Roder et al., 1996, 1999; Hotting and Roder, 2004; Collignon et al., 2006). Since these studies demonstrated that blind and sighted participants did not differ in sensory sensitivity on these tasks, these findings appear to reflect superior attentional capabilities (Cattaneo and Vecchi, 2011), although teasing apart attentional and perceptual deficits is often difficult. Blindness is also associated with superior performance on tasks of *divided* attention, such as dichotic listening (Hugdahl et al., 2004), and tasks requiring dividing attention across more than one sensory modality (Kujala et al., 1995). Again, such a pattern of performance contrasts sharply with what has been long been observed in schizophrenia, namely, severely impaired selective and divided auditory attention (Bleuler, 1950; Green et al., 1994; Heinrichs and Zakzanis, 1998; Bruder et al., 1999; Light and Braff, 2005a,b; Scholes and Martin-Iverson, 2010). In short, the improvements in sensory, perceptual, and attentional auditory functioning in C/E blindness represent changes that appear to essentially eliminate the possibility of the development of the set of auditory impairments and their consequences that form an important component of schizophrenia.

### Working and long-term memory

Compared to healthy, sighted individuals, C/E blind individuals have superior working memory capacity, in terms of both the amount that can be processed, and the ability to recall the sequence in which information was presented (Hull and Mason, 1995; Amedi et al., 2003; Roder and Neville, 2003; Raz et al., 2007). The authors of a recent study that matched perceptual threshold of blind and sighted subjects concluded that superior short-term memory in C/E blind people was due to enhancement of stimulus encoding (related to the processes reviewed above), rather than to differences in storage or recall processes (Rokem and Ahissar, 2009). In addition, it is thought that, in C/E blindness, primary reliance on controlled, sequential processes (i.e., via haptic and auditory perception) during navigation – as opposed to the more automatic and parallel processing normally afforded by vision – leads to superior working memory abilities (Raz et al., 2007; Salillas et al., 2009; Cattaneo and Vecchi, 2011). C/E blind people have also demonstrated superior long-term memory compared to sighted controls (Bull et al., 1983; Roder et al., 2001). All of this is in stark contrast to schizophrenia, in which working memory (Silver et al., 2003; Conklin et al., 2005; Brahmbhatt et al., 2006; Horan et al., 2008; Silverstein et al., 2010) and long-term memory (Van Snellenberg, 2009) impairments are typically observed, along with problems in stimulus organization during encoding (Harvey et al., 1986; Brebion et al., 1997, 2004; Landgraf et al., 2011).

These data have important clinical implications. A critical function of WM is to integrate perceptual information with information stored in long-term memory, to allow for efficient learning, memory, and reasoning (Cattaneo and Vecchi, 2011). Enhanced WM abilities would thus facilitate these other processes, as is the case in C/E blindness (Cattaneo and Vecchi, 2011), whereas impairment would have the opposite effect, as is the case in schizophrenia (Tek et al., 2002). Thus, it can again be seen how C/E blindness could protect against development of the cognitive impairments that comprise the syndrome of schizophrenia.

### Language

Congenital or early blind children often experience language delays and abnormalities related to semantic, syntactic, and phonological processing (Perez-Pereira and Conti-Ramsden, 1999). One possible consequence of these is a reduction in the risk of developing disordered thought, which is commonly seen in schizophrenia. For example, many thought-disordered patients with schizophrenia have a tendency toward overabstraction and overinclusion in their thinking, as well as overly elaborated (and more easily primed) semantic networks (Siekmeier and Hoffman, 2002; Lerner et al., 2012). In addition, compared to healthy controls, individuals with schizophrenia more often use language in odd ways, including generating neologisms (i.e., novel words; Solovay et al., 1987; Kreher et al., 2008). In contrast, it has been shown that compared to sighted children, blind children are characterized by a lack of overgeneralization of concepts and categories, and a reduced number of word inventions (Andersen et al., 1984, 1993). It has also been shown that semantic networks in blind children are more dependent on language, and less dependent on sensory experience, than sighted children (Pring, 1988). This could be a protective factor against delusion formation secondary to altered sensory experience, as has been hypothesized to exist in schizophrenia (Maher, 1974; Uhlhaas and Mishara, 2007).

### Olfaction

Cuevas et al. (2009) demonstrated that congenitally blind males were better at discriminating odors, and at naming familiar odors, compared to sighted subjects. Other studies have reported mixed results, for example, demonstrating superior odor identification but not odor sensitivity (Wakefield et al., 2004). However, studies with mixed findings have grouped early blind and late-blind subjects together, and did not match groups on gender (women are typically better than men at olfaction tasks; Cattaneo and Vecchi, 2011). While more research is needed on this issue, evidence does suggest that people born blind may develop increased olfactory abilities (Kupers et al., 2011). This is in contrast to people with schizophrenia, in which olfactory impairments have been reported (Nguyen et al., 2010; Kamath et al., 2011). Although the functional consequences of olfactory impairment in schizophrenia are not well understood, such deficits could impair self-regulation, learning, attachment, and interpersonal functioning (see Sullivan, 2003). For example, the detection of maternal odor can promote attachment to the mother, reduce stress, and help normalize the sleep-wake cycle in infants (Goodin-Jones et al., 1997; Sullivan and Toubas, 1998). In addition, odor perception is important in forming bonds with peers in childhood (Sullivan, 2000). It is therefore interesting that risk for schizophrenia is associated with problems in maternal attachment (demonstrated at 3 days, 1, and 6 years of age; Naslund et al., 1984; Persson-Blennow et al., 1984; McNeil and Kaij, 1987), increased stress reactivity (Myin-Germeys and van Os, 2007), and fewer peer relationships (Schenkel and Silverstein, 2004), as well as olfactory processing deficits (Brewer et al., 2003).

## Construction of Subjective Experience

In this section, we discuss: (1) differences between people who are C/E blind and people with schizophrenia with regard to the relative importance of parallel versus serial data acquisition in the generation and quality of subjective experience; and (2) the role that preservation of a form of visual imagery in C/E blindness may play in the integration of experience. As noted above, C/E blind people rely to a much greater extent than sighted people on serial processing due to primary reliance on haptic and auditory processing, and lack of visual input. That is, due to the temporal-sequential nature of data acquisition about objects in auditory and haptic perception, the relatively smaller amounts of information available at any one time in haptic perception compared to vision (Barber and Lederman, 1988; Amadeo and Speicher, 1995; Herssens, 2010), and the more rapidly changing nature of information flow in audition relative to vision (Shamma et al., 2011), formation of coherent mental representations in haptics and audition requires that information must be “built up” over time to a greater extent than in vision (Geenens, 1999). One result of this imposed learning style is that C/E blind individuals develop abilities in sequential and controlled processing that may be superior to those of sighted people (Vecchi et al., 2004; Raz et al., 2007). In addition, as we argue below, compensatory processes involving working memory capacity, perceptual organization via controlled processing, multisensory integration, learning, and a preserved form of visual imagery lead to a rich, seemingly automatic, and non-fragmented flow of experience (see Cattaneo et al., 2008; Salillas et al., 2009 and above; Cattaneo and Vecchi, 2011). In contrast, in schizophrenia, impairments in working memory capacity, perceptual organization, and multisensory integration, which develop long after birth and for which there appear to be no compensation, contribute to an over-reliance on serial processing that is often inefficient, and that can lead to fragmented subjective experience (Chapman, 1966; Knight, 1984; Carr and Wale, 1986; Knight and Silverstein, 1998; Silverstein and Keane, 2011a). This is illustrated in the following quote from a person with schizophrenia: “Everything I see is split up. It’s like a photograph that’s torn in bits and put together again. If somebody moves or speaks, everything I see disappears quickly and I have to put it together again” (Chapman, 1966, p. 229).

Interesting examples of how blind individuals construct mental representations incrementally via haptic perception come from Helen Keller’s (1908) book *The World I Live In*; her descriptions also illustrate how this process can be facilitated by prior knowledge and augmented with imagery. For example, she noted: “My fingers cannot, of course, get the impression of a large whole at a glance; but I feel the parts, and my mind puts them together” (p. 7). Similarly: “My hand has its share in this multiple knowledge, but it must never be forgotten that with the fingers I see only a very small portion of a surface, and that I must pass my hand continually over it before my touch grasps the whole. It is still more important, however, to remember that my imagination is not tethered to certain points, locations, and distances. It puts all the parts together simultaneously as if it saw or knew instead of feeling them.” (pp. 28–29). It is true of course that vision also incrementally builds distal representations, viz, through saccades or shifts in attention (Ullman, 1984), but because this process is not limited by the speed of grasping and reaching, it plausibly occurs over a much shorter time scale, lessening the load on working memory. In short, while C/E blindness is characterized by a greater than normal reliance on serial processing, compensatory processes lead to superior serial processing abilities and to integrated subjective experience.

In addition, we speculate that a preserved form of visual imagery is involved in the spatial integration abilities of C/E blind people. A reasonable amount of evidence supports the presence of imagery in C/E blindness, including: (1) visual cortex areas are activated by input from other senses in blind and non-blind people (Amedi et al., 2001, 2002; Ortiz et al., 2011); (2) the occipital lobe helps generate mental representations even when there has been no prior visual input (Amedi et al., 2008); (3) after training with tactile stimulation, blind people reported visual qualia, and the extent of these reports were related to ERP activity in the lateral occipital complex (Ortiz et al., 2011); (4) C/E blind people have visual content in their dreams, and can draw it, and the extent of their dream imagery is negatively correlated with EEG alpha band power, just as it is in sighted individuals (Bertolo et al., 2003); (5) C/E blind people report visual imagery (Cornoldi et al., 1979; Zimler and Keenan, 1983), although this is reduced, and more verbally mediated, compared to sighted people (Cornoldi et al., 1979); (6) however, spatial representation is not completely verbally mediated in blind people; during the encoding and working memory maintenance phases of a haptic mental rotation task, both sighted and blind subjects demonstrated increased parietal activation (as demonstrated in ERP data; Roder et al., 1997); (7) spatial images are supramodal, in that they are not simply the sum of modality-specific inputs; (8) there are several characteristics of visual experience, such as size, contour, and edge information, shape and texture that can be perceived through touch, and blind people can generate visual representations of haptically explored objects (reviewed in Cattaneo et al., 2008; Cattaneo and Vecchi, 2011); (9) vision and haptics may share common representations (Aleman et al., 2001); and (10) both the dorsal and ventral pathways are thought to contain supramodal regions to code shape and location of objects, respectively, regardless of the nature of the sensory input (Pietrini et al., 2009). We suggest, based on the quotes cited above, and others in the literature, that these “visual” experiences and supramodal representations aid in integrating subjective experience in C/E blindness. However, direct evidence for this claim has not yet been reported.

## C/E Blindness is Protected from Negative Developmental Effects of Visual Processing Impairments

Vision allows for the simultaneous perception of a great deal of information, and therefore for attention to be spread over many stimuli, or to be switched between stimuli. In schizophrenia, with its related sensory gating deficits, this can lead to the experience of being flooded with stimuli, and of stimuli being more intense than usual (Carr and Wale, 1986). In contrast, blindness is associated with touch as the primary method of exploring the environment. The nature of haptic perception is such that the person can only attend to the object currently being touched, and in this way attention cannot be involuntarily drawn to other objects (unless there are auditory or olfactory cues to other objects; Cattaneo and Vecchi, 2011).

Of course, we are not saying that vision is, by itself, a risk factor for schizophrenia. However, in combination with sensory gating, perceptual organization, and working memory impairments, visual processing, with its parallel inputs, may contribute to sensory flooding and subsequent disorganized cognition and behavior in schizophrenia. That is, visual *impairment* may be a risk factor for schizophrenia, not only in being reflective of abnormal neural development but also by being a cause of it. Conversely, C/E blindness may exert its protective effects against schizophrenia, in part, by preventing the possibility of abnormal visual input. Evidence consistent with this comes from several sources, including: (1) visual dysfunction in children of mothers with schizophrenia predicts the later development of schizophrenia (Schubert et al., 2005); (2) children (regardless of parental history) who later developed schizophrenia-spectrum disorders had significantly higher eye exam scale and strabismus scale scores compared to children who developed other non-psychotic psychopathology and children who did not develop a mental illness, while functioning in other sensory domains was far less impaired (Schiffman et al., 2006); and (3) the high specificity of visual abnormalities for predicting transition to schizophrenia among at-risk youth (Klosterkotter, 1992). In all of these cases, even if the presence of visual dysfunction is a reflection of the diathesis for schizophrenia rather than a primary cause of it, the continued state of abnormal visual input may lead to further perceptual and cognitive problems throughout development that increase the risk of developing the full-blown disorder.

Impaired visual functioning may also compromise multisensory integration, which, in turn, may lead to other cognitive deficits in schizophrenia. Efficient integration of different sensory inputs requires remapping these into an external spatial reference frame (Roder et al., 2007), which requires normal vision to properly develop (Collignon et al., 2009). However, in schizophrenia, there are multiple visual processing impairments (e.g., in gain control, perceptual organization, motion perception, etc.; Butler et al., 2008). Multisensory integration is also impaired in schizophrenia (Williams et al., 2010), and it is thought that deficits in visual processing contribute to this (Landgraf et al., 2012). In contrast, blind people appear to rely on somatotopic spatial coordinates as opposed to external, visual, coordinates (Roder et al., 2004). Therefore, in contrast to schizophrenia, in C/E blindness, the primary sense used to create a reference frame for multisensory integration (i.e., touch) is associated with superior perceptual abilities compared to sighted people.

Blind people rely primarily on vestibular and somatosensory feedback for motor control. This leads to enhanced voluntary motor control and motor-kinesthetic processing (Deutschlander et al., 2009). In contrast, schizophrenia is associated with impaired motor control (Cortese et al., 2005; Putzhammer and Klein, 2006; Velasques et al., 2011), and it has been hypothesized that this could be related to passivity phenomena and the development of delusions of control (e.g., the belief that one is not the agent of one’s own actions, and/or that an external entity is controlling one’s thoughts and/or actions; Danckert et al., 2004). Abnormalities in movement have also been observed in people at high risk for schizophrenia (Mittal et al., 2008). Therefore, the enhanced development of motor control and kinesthetic processing in C/E blindness can be viewed as protective against these schizophrenia-related symptom formation processes.

Finally, it has been recently proposed that visual processing disturbances in individuals at-risk for schizophrenia may contribute to the later development of the disorder by causing impairments in body perception, involving the senses of “ego boundaries,” body ownership, body agency, and sense of self in space. These phenomena may then lead to problems in sense of identity, and in awareness of symptoms and insight into illness (Landgraf et al., 2012). Body ownership and agency can be measured experimentally with the “rubber hand illusion” (Ehrsson et al., 2004). In this paradigm, subjects have the experience that they are touching their own right hand with their left index finger, when in reality they are touching a rubber hand with their left index finger while the experimenter touches their right hand in a synchronized manner. This effect is enhanced in schizophrenia and is thought to reflect abnormal body perception including a reduction in sense of body ownership (Thakkar et al., 2011). In contrast, in people with C/E blindness, the illusion is not experienced (Petkova et al., 2012). These results have been interpreted as indicating that C/E blind people have a more veridical perception of self-touch, along with a *less* flexible and dynamic representation of their own body in space compared to sighted individuals (Petkova et al., 2012). We believe that this reduced flexibility in dynamic representation of the body, secondary to loss of vision, is a protective factor against the body perception abnormalities associated with schizophrenia that were noted above.

## Neuroplasticity

In this section we review evidence on how and where in the brain changes occur in response to C/E blindness, and how these subserve the changes in cognitive functioning discussed above. There is a large literature on brain plasticity in blind people, including evidence for developmental crossmodal plasticity (see below) – where areas of the occipital lobe are recruited for auditory and haptic perception (see Cattaneo and Vecchi, 2011 for a review), and also for olfaction (Kupers et al., 2011). Beyond this, however, other changes in developmental *intra*modal plasticity can occur which appear to result in perceptual and cognitive abilities that are opposite to those found in schizophrenia. For example, a magnetic source localization study (Elbert et al., 2002) indicated that dipoles associated with processing low- and high-frequency tones were more distant in blind people than in sighted subjects, suggesting an expansion of regions in the auditory cortex, supporting the superior pitch discrimination abilities noted above. Further evidence for intramodal plasticity in blind individuals is *reduced* hemodynamic responses to auditory stimulation in comparison to sighted subjects, which is thought to reflect *increased* efficiency in auditory signal processing within the temporal lobe, in light of the generally superior auditory processing abilities in this population (Stevens and Weaver, 2009). Evidence for changes regarding haptic perception comes from findings of *reduced* accuracy in identifying which finger is touched during a sensory threshold task (Sterr et al., 1998, 2003), within the context of overall superior tactile perception (Wong et al., 2011). These data suggested altered cortical representations for touch in the sense that the cortical representations of individual fingers are “fused” together due to their simultaneous use during Braille reading. Moreover, these data are strongest for the hand used to read Braille, and do not generalize to other body parts that are not used for reading, such as lips. Of note, the “holistic” processing of Braille letter patterns and reduced precision of individual finger-level input is in contrast to what is often observed in the visual processing of people with schizophrenia, where processing of holistic visual patterns is impaired, sometimes with enhanced processing of individual visual details (reviewed in Uhlhaas and Silverstein, 2005; Silverstein and Keane, 2011a).

Developmental crossmodal plasticity can lead to superior performance compared to sighted subjects, and there are several interesting instances of this relevant to schizophrenia. For example, somatosensory and auditory ERP data from oddball and MMN tasks (which involve detection of a deviation from a sequential pattern in an attended or unattended sequence of stimuli, respectively), indicate normal ERP amplitude, with more activity over the occipital cortex in blind subjects, and enhanced pre-attentional processing in blind compared to sighted subjects (Kujala et al., 1995; Roder et al., 1996). This is in marked contrast to abnormal ERP amplitudes during oddball and MMN tasks in people with schizophrenia (e.g., Kayser et al., 2001) as noted above. Aside from developmental neuroplasticity, other ERP data from oddball tasks in people with schizophrenia suggested reduced short-term neuroplasticity. For example, when people with schizophrenia are presented with a high-frequency sequence of individual auditory stimulations, their ERP signatures reveal abnormally reduced long-term potentiation (Mears and Spencer, 2012). In contrast, short-term neuroplasticity, at least involving the occipital cortex, may be greater in people with C/E blindness (De Volder et al., 1999).

A major focus of research on visual processing in schizophrenia is impaired functioning in the dorsal and ventral visual streams. Therefore, we briefly summarize evidence that, in contrast to what is observed in schizophrenia, functioning in these pathways is intact in C/E blindness, *despite the absence of visual input*. Much evidence suggests that the dorsal stream is specialized for determining where an object is and for guiding action, whereas the ventral stream is specialized for analyzing form and color for determining what an object is (Goodale, 1993; Goodale et al., 2005). Many studies indicate impaired functioning of both of these streams in schizophrenia (e.g., King et al., 2008; Silverstein et al., 2009; Sehatpour et al., 2010; Lalor et al., 2012; Plomp et al., 2012). Surprisingly, these pathways appear to subserve similar functions in blind people as they do in psychiatrically healthy sighted people, suggesting that they can develop even in the absence of visual experience, and therefore that their representations are supramodal (Cattaneo and Vecchi, 2011; Ptito et al., 2012). For example, during spatial imagery tasks using auditory or tactile stimuli, early blind and sighted individuals demonstrated similar dorsal stream and frontal eye field activation, as assessed via PET or fMRI (Vanlierde et al., 2003; Garg et al., 2007; Bonino et al., 2008). In addition, dorsal areas devoted to processing motion information in sighted people (e.g., V5/MT) are employed in blind people in response to tactile and auditory motion cues (e.g., Ptito et al., 2009; Bedny et al., 2010; Matteau et al., 2010). These areas are notably underactivated during perception of visual motion in schizophrenia, in the context of impaired motion perception (Chen, 2011). In the ventral stream, blind subjects demonstrated similar activation during tactile object perception to that shown by sighted subjects during visual and haptic exploration of the same objects (Pietrini et al., 2004; Ptito et al., 2012).

It should be noted that while occipital lobe activity can occur in sighted subjects during tactile and auditory processing tasks, this has been found to be due to visual imagery (Sathian, 2005), which activates early visual cortex areas in a retinotopic fashion (Slotnick et al., 2005), and to calibration of head-centered sound localization (Zimmer et al., 2004). In contrast, occipital cortex activity in C/E blind subjects appears to support actual non-visual stimulus processing, and to represent a true crossmodal reorganization (see Roder et al., 1997; Cattaneo and Vecchi, 2011 for a review). Consistent with this, transcranial magnetic stimulation (TMS), applied to the occipital cortex, disrupts Braille reading ability in blind individuals, but not in sighted individuals, whereas the reverse is true when TMS is applied over the somatosensory cortex in the parietal lobe (Cohen et al., 1997). Similarly, TMS over the occipital cortex induces tactile sensations in blind readers’ fingers, but only phosphene perception for sighted subjects (Ptito et al., 2008).

As noted above, schizophrenia patients have demonstrated olfactory deficits in several studies, while C/E blind individuals have demonstrated superior olfactory abilities. A recent study, comparing congenitally blind subjects to blindfolded sighted controls, demonstrated that this is due to enhanced processing in areas normally dedicated to olfaction (e.g., amygdala, hippocampus) *and* in multiple regions of the occipital cortex (Kupers et al., 2011). This is further evidence of both the supramodal nature of representations in the C/E visually deprived occipital lobe, and also of crossmodal plasticity in C/E blindness.

In addition to subserving enhanced non-visual sensory processing, fMRI data indicate that the occipital lobe in C/E blind individuals also supports syntactic and semantic (more so than phonological) aspects of language processing (Roder et al., 2002; Burton et al., 2003), as well as verbal memory, episodic memory, and verbal fluency (Amedi et al., 2003; Raz et al., 2005), all of these being processes that are impaired in schizophrenia (Tendolkar et al., 2002; Fisher et al., 2009; Costafreda et al., 2011). For example, in addition to occipital lobe activation during memory retrieval, and a correlation between occipital activation and episodic memory in blind but not sighted subjects (Raz et al., 2005), repetitive transcranial magnetic stimulation (rTMS) over the occipital lobe impaired performance in a verb generation task for blind but not sighted subjects (Amedi et al., 2004).

Another consequence of blindness may be increased communication between brain regions. Several studies have demonstrated increased effective connectivity between the occipital lobe and temporal and prefrontal regions in early blind individuals (e.g., Noppeney et al., 2003; Leclerc et al., 2005). This is in contrast to schizophrenia, where reduced connectivity is typically found (e.g., Kim et al., 2008; Dima et al., 2009, 2010), including between occipital and temporal and frontal regions (Rigucci et al., 2012).

Finally, Sanders et al. (2003), based on evidence of visual cortex plasticity in dark-reared animals (Chen and Bear, 2007; Chen et al., 2007), proposed that an effect of early blindness is increased NMDA-receptor activity (although accompanied by reduced numbers of receptors) in visual cortex, and in regions with which this has many connections, primarily the anterior cingulate cortex (ACC), which is involved in resolving response competition and context sensitivity, two cognitive domains that are impaired in schizophrenia (Cohen and Servan-Schreiber, 1992; Dolan et al., 1995; Phillips and Silverstein, 2003). Moreover, schizophrenia has been characterized by NMDA-receptor hypofunction (Phillips and Silverstein, 2003), and, in numerous studies, by ACC hypoactivation (Dolan et al., 1995). The consequences of NMDA-receptor hypofunction in schizophrenia have been hypothesized to include impairments in sensory gating, perception, working memory, learning, and the full range of positive and negative symptoms (Phillips and Silverstein, 2003; Coyle, 2012). Therefore, an increase in NMDA-receptor activity in C/E blindness could have widespread positive effects on cognition, and protective effects against symptoms.

In short, there are several ways in which the neuroplasticity that results from C/E blindness results in changes that are opposite to those observed in schizophrenia, and that may represent protective effects. Specifically, the evidence reviewed above suggests that, in C/E blindness: (1) despite the loss of visual input, cortical reorganization during early development leads to accurate form and space perception (via auditory and tactile processing) which is supported by brain areas that function abnormally in schizophrenia (e.g., dorsal and ventral visual streams); (2) this developmental plasticity also leads to use of occipital areas for functions such as preattentive processing (e.g., as reflected in the MMN), language, memory, and olfaction – all of which are impaired in schizophrenia – which may underlie the superior functioning in those areas in C/E blindness reviewed above; and (3) in addition to these sequelae of developmental plasticity in C/E blindness, there is also evidence of normal later-onset plasticity, which may in part reflect prolonged plasticity of the occipital cortex in C/E blindness (De Volder et al., 1999), and which is the basis for the success of sensory substitution devices (Ward and Meijer, 2010). In contrast, later-onset plasticity has been shown to be reduced in schizophrenia in several domains (Fitzgerald et al., 2004; Oxley et al., 2004; Daskalakis et al., 2008; Hasan et al., 2011, 2012; Mears and Spencer, 2012).

## Congenital or Early Blindness is Not Protective Against Mental Disorders in General

While C/E blindness may protect against schizophrenia, they do not protect against mental illness in general. A range of mental and behavioral disorders has been reported in blind people. For example, anxiety levels have been reported to be higher in congenitally blind than sighted adolescents (Bolat et al., 2011), and autistic symptoms and autism are common in blind children (Keeler, 1958; Wing, 1969; Chess, 1971; Chase, 1972; Fraiberg, 1977; Rogers and Newhart-Larson, 1989; Brown et al., 1997; Ek et al., 1998; Carvill, 2001). Sharp (1993) reported a case of anorexia nervosa and depression in a woman blind since the age of 9 months. A recent case study (Kocourkova et al., 2011) described a case of an adolescent patient, blind from early childhood, who showed symptoms of anorexia, depression, suicidal behavior, and self-harming. A conclusion in this report was that body image is a mental construct that is not dependent on sensory perception. Finally, Musial et al. (2007) reported a case of arachnophobia in a congenitally blind person, suggesting that visual cues are not necessary for the development of phobic disorders.

## Congenital Deafblindness is Not Protective Against Schizophrenia

Protective effects of blindness may not exist if other forms of severe sensory loss are present. For example, congenital deafblindness is associated with elevated rates of psychosis, as well as mental retardation (Dammeyer, 2011). Also, children with Usher syndrome, in which people are typically deaf from birth and then develop retinitis pigmentosa leading to blindness in childhood, has been associated with psychotic symptoms and schizophrenia (Dammeyer, 2012). The reason why adding deafness to blindness removes the apparent effect of the latter against schizophrenia is not clear at present. One possibility is that blindness by itself presents a surmountable challenge to cope with the environment and thereby fosters compensatory sensory, perceptual, and cognitive changes that lead to a surprisingly high level of functioning. Deafblindness, on the other hand, may so seriously restrict the opportunity for environmental interaction that it also stunts the development of cognitively based coping strategies. For example, congenital deafblindness typically leads to significant delays in achieving several cognitive milestones, and to profound impairments in speed of processing, the ability to integrate disparate items of information, attention, communication, and the capacity for symbolic understanding and expression (Geenens, 1999; Bruce, 2005; Shamma et al., 2011). It is worth noting here that although C/E blindness and C/E deafblindness are both relatively rare, there are no published cases of the former being comorbid with schizophrenia, whereas there are many reported cases of this comorbidity with the latter.

## Sensory Loss in General Does Not Protect Against Schizophrenia

Another important consideration is that sensory loss *per se* does not protect against schizophrenia. For example, deafness occurs in schizophrenia at as high a rate as in the general population (Kitson and Fry, 1990), and congenital deafness is a risk factor for the development of psychotic symptoms (Thewissen et al., 2005; Atkinson, 2006), including, interestingly, higher rates of visual and somatic hallucinations than are found in the non-deaf people with schizophrenia (Cutting, 1985), which often co-occur with reports of hearing voices (Du Feu and McKenna, 1999). And, while deafness may be associated with compensatory cognitive changes (see Bross, 1979; Dye and Bavelier, 2010; e.g., heightened attention to visual peripheral cues in people who use sign language; Proksch and Bavelier, 2002), these appear to not protect against schizophrenia, although they may lead to subtle differences in profiles of cognitive impairment between deaf and hearing people with schizophrenia (Horton and Silverstein, 2007, 2011). Moreover, in some cases, compensatory changes may not develop until adulthood (Rettenbach et al., 1999).

## Mediating Biological Variables

The hypothesized protective effects of blindness on schizophrenia may be mediated by other factors. For example, Horrobin (1979) proposed that abnormal pineal gland function and resulting prostaglandin activity are etiological factors in schizophrenia. This is supported by evidence of increased inflammation and reduced melatonin levels in schizophrenia patients (Anderson and Maes, 2012; Muller et al., 2012). Conversely, blind individuals have elevated melatonin levels (Bellastella et al., 1995), which has been shown to attenuate cognitive impairments (Peck et al., 2004) and reduce inflammation (Cuzzocrea et al., 1999; Sharma et al., 2012). Another potential mechanism involves cortical thickness and pruning. Schizophrenia is typically characterized by cortical thinning (Cobia et al., 2012), and it has been hypothesized that excessive neuronal pruning takes place during adolescence (Boksa, 2012). In contrast, the visual cortex of C/E blind people – which as noted above is involved in multiple perceptual and cognitive processes – is thicker than in sighted people, and is thought to be characterized by a less than normal amount of pruning, due to deprivation of visual experience (Jiang et al., 2009). A consequence of this thickening is that, even if genes related to schizophrenia cause excessive pruning in someone with C/E blindness, the remaining number of neurons may still be greater than normal in some areas. This, in combination with the cortical reorganization noted above, leading to occipital regions being used for non-visual cognitive processes, may protect blind people against crossing the threshold needed for psychotic symptoms to emerge (i.e., an adequate number of neurons devoted to specific types of processing may be present even if pruning is excessive).

## Conclusion

This paper builds on previous reports of a lack of schizophrenia in people with C/E blindness by advancing the hypothesis that the latter alters cognition and fosters neuroplasticity in ways that confer protective effects against schizophrenia. The reviewed evidence indicates that: (1) schizophrenia is primarily a cognitive disorder; (2) many of the cognitive functions that are impaired in schizophrenia are enhanced among the C/E blind; (3) C/E blindness involves *reduced* flexibility in language and in dynamic representation of the body, and these reductions may protect against thought disorder and alterations in experience of the self, respectively; (4) the mechanisms noted in 2 and 3 above are significantly less affected if the onset of blindness is after the first few months of life; (5) other forms of C/E sensory loss do not protect against schizophrenia; and (6) C/E blindness does not protect against other disorders, suggesting that there is a special link between schizophrenia and visual processing.

Of course, schizophrenia is characterized by cognitive impairments in addition to those described here, such as in executive functioning, that are thought to rely heavily on functioning of the dorsolateral prefrontal cortex (DLPFC). There appears to be almost no research comparing blind (congenital or acquired) and sighted people on these functions, however, and so whether blindness confers any protective effects on them remains an open question. Note, however, that the DLPFC does not fully develop until late adolescence or early adulthood. In contrast, cognitive and academic decline, and interpersonal dysfunction, typically begin in late childhood or early adolescence in people who go on to develop schizophrenia, and this age range corresponds to the time frame in which the visual system in humans is most plastic (i.e., until ∼14–16 years of age; Cohen et al., 1999). This suggests that the protective sequelae of C/E blindness involve changes in more basic sensory, perceptual, and cognitive (i.e., non-executive) functions.

While the idea of one abnormal condition having a protective effect against another condition may seem unusual, it is not unknown. For example, several studies have now replicated the finding that schizophrenia appears to have an inverse relationship with rheumatoid arthritis (RA), with some estimates putting the risk for RA among schizophrenia patients at 30% that of control counterparts (Eaton et al., 1992; Mors et al., 1999; Oken and Schulzer, 1999). Conversely, there may also be a reduced risk for developing schizophrenia among individuals with RA (Gorwood et al., 2004). This is especially low considering smoking is a risk factor for developing RA (Stolt et al., 2003) and that a disproportionally large percentage (60–90%) of individuals with schizophrenia smoke (Dervaux and Laqueille, 2008). As noted, we hypothesize that the cognitive benefits conferred by C/E blindness act as a prophylactic for schizophrenia but not other mental disorders. This hypothesis, if confirmed, has implications for determining when cognitive training should occur for people with, or at-risk for, schizophrenia, and for the nature of such training. It also has implications for whether insights from C/E blindness can be used to design interventions to prevent schizophrenia. Each of these issues will be addressed in turn below.

Evidence from animal studies indicates that prevention of schizophrenia-related cognitive impairment can be achieved by early cognitive training. For example, in one study, rats received a ventral hippocampus lesion that induces a spatial working memory deficit similar to that found among schizophrenia patients. It was found that among animals that had been provided with pre-lesioning cognitive training, the typical post-lesion working memory deficit did not arise (Lee et al., 2012). While similar preventive data do not exist in humans, a recent study of cognitive remediation in first-episode schizophrenia demonstrated that it significantly slowed the typical gray matter loss associated with the first years after an initial psychotic episode (Eack et al., 2010). Therefore, it is reasonable to study whether the long-term effects of cognitive training in people at high risk for the disorder could be at least as strong, if not stronger, than effects of cognitive training introduced after a diagnosis of schizophrenia is made, given that the effects are occurring in the context of a healthier and younger brain.

But when should training occur? Evidence indicates that intervening when people first begin to show psychotic symptoms or functional decline (i.e., in the “ultra high-risk” state) can delay, but does not prevent, schizophrenia (Yung and Nelson, 2011). Therefore, it has recently been proposed that intervening at a “pluripotent risk stage” – the time of the earliest emergence of behavioral and cognitive difficulties (typically 9–13 years of age) that are non-specific as to final diagnosis – would be more effective (Agius et al., 2010; McGorry, 2010; McGorry et al., 2010; Morgan et al., in press). Whether or not such a strategy can prevent schizophrenia, it may be an ideal time for intensive cognitive training, in terms of affording protection against later and severe schizophrenia-related cognitive impairment. Of course, even earlier training could be effective, but the feasibility of identifying people at high risk for mental illness at younger than 9-years old has not been well-established, except in cases where an identical twin or first-degree relative has the disorder.

What sort of training would be effective? Based on the evidence reviewed above, we suggest that cognitive training in young people at-risk for schizophrenia should be *comprehensive in nature*, and should focus to a greater degree than it has in the past on sensory and perceptual functioning. For example, in addition to a focus on improving aspects of attention, it should focus on improving sensory tuning and the accuracy of sensory representations, perceptual organization, multisensory integration, and controlled processing including integration of bottom-up sensory data with stored information (see Heller quotes above, and Hemsley, 1996). Some interventions have already demonstrated success in improving functioning in these specific areas in humans (Temple et al., 2003; Mahncke et al., 2006; Genevsky et al., 2010) and animals (Polley et al., 2006; Zhou et al., 2011). In addition, although schizophrenia patients often demonstrate reduced plasticity, it is not absent, and capacity for perceptual and cognitive change has been demonstrated (Fisher et al., 2009, 2010; Silverstein and Keane, 2009) both in and outside the context of specific interventions. Therefore, it is possible that even greater plasticity can be harnessed earlier in the developmental course of the illness, before psychotic symptoms emerge and near the onset of, or prior to, cognitive and functional decline. In addition, it has already been demonstrated in schizophrenia that improving auditory precision by repeated practicing of tone discrimination leads to improvements in auditory and (more interestingly) higher level cognitive functions (e.g., verbal learning and memory; Adcock et al., 2009; Fisher et al., 2009). Therefore, broader improvements (beyond the trained task) may occur in at-risk youth with similar practice.

An additional strategy for people at-risk for schizophrenia involves increasing reliance on non-visual sensory modalities to enhance their functioning. Already it has been shown in people with established schizophrenia, for example, that steady-state auditory responses, which are typically abnormally reduced and associated with an increase in broadband noise, can be significantly improved, and to a greater degree than occurs in healthy people, by closing the eyes during task performance (Griskova-Bulanova et al., 2012). Fostering cognitive reserve, and trying to prevent schizophrenia, or at least minimize later illness-related cognitive impairment, by strengthening capacity and efficiency in non-visual domains is an as yet untested strategy. However, the goal of creating a schizophrenia-specific, *acquired* cognitive reserve, in a manner guided by compensatory changes observed in C/E blindness, would appear to be a reasonable and promising direction to pursue, given that premorbid cognitive reserve predicts later cognitive functioning in normal aging (Steffener and Stern, 2012), neurodegenerative disorders (Koerts et al., 2012; Stern, 2012), and schizophrenia (de La Serna et al., 2013).

Can an understanding of cognition and neuroplasticity in C/E blindness be used to reduce the likelihood of schizophrenia? At first glance, the answer seems to be “no” since: (1) only C/E blindness (but not blindness acquired after the first few months of life) is associated with a protective effect; (2) it is difficult to identify most people who will develop schizophrenia during the first year of life; (3) it is not clear what type of training could be given to people less than 1-year old, given limited attention span and undeveloped verbal abilities; and (4) the presence of vision is likely to reduce the extent of developmental neuroplasticity. However, the ultimate answer to this question may not be this simple. For example, much remains to be learned about biological and computational changes associated with C/E, and later-developing, blindness, and so further investigation into these issues may reveal clues for intervention development. Also, it is not known beyond what age fostering neuroplasticity and cognitive enhancement might no longer be able to prevent schizophrenia, but only reduce schizophrenia-related cognitive impairment. Third, since it is not C/E blindness *per se*, but rather, the corresponding brain and cognitive changes that are protective against schizophrenia, it is possible that methods other than naturally occurring C/E blindness can confer protective effects. For example, an open question is the extent to which particularly intensive sensory-perceptual-cognitive interventions delivered to at-risk youth past the first year of life can create some of the protective changes associated with naturally occurring C/E blindness.

## Conflict of Interest Statement

The authors declare that the research was conducted in the absence of any commercial or financial relationships that could be construed as a potential conflict of interest.
